# Solitary adrenal metastatic adenocarcinoma one year after radical gastrectomy for gastric cancer: a case report

**DOI:** 10.3389/fmed.2026.1795557

**Published:** 2026-03-19

**Authors:** Yingming Song, Jiahui Wu, Zhengyi Jin

**Affiliations:** 1 Department of Gastrointestinal Surgery, Heping Hospital Affiliated to Changzhi Medical College, Changzhi, China; 2 The First Clinical College of Changzhi Medical College, Changzhi, China

**Keywords:** adrenal metastasis, case report, gastric cancer, immunochemotherapy, solitary adrenal tumor

## Abstract

**Background:**

Adrenal metastasis from gastric cancer is rare and usually occurs as part of widespread systemic dissemination. Solitary adrenal metastasis detected during postoperative follow-up is extremely uncommon and easily overlooked due to nonspecific clinical manifestations.

**Case presentation:**

We report the case of a 69-year-old man who underwent radical gastrectomy for esophagogastric junction cancer one year earlier. Histopathology revealed diffuse infiltrative mucinous adenocarcinoma with components of signet-ring cell carcinoma and neuroendocrine carcinoma (pT4N3Mx). The patient completed six cycles of adjuvant chemotherapy with tegafur–gimeracil–oteracil (S-1) plus oxaliplatin, and routine follow-up examinations showed no evidence of recurrence. Twenty-one months postoperatively, he presented with left upper abdominal pain. Computed tomography revealed a solitary mass in the left adrenal gland. Tumor markers, including CA72-4, CA19-9, and carcinoembryonic antigen, were markedly elevated. Ultrasound-guided biopsy confirmed metastatic adenocarcinoma of gastric origin. The patient was treated with SOX chemotherapy combined with the PD-1 inhibitor tislelizumab. After the first treatment cycle, his general condition improved, and he remains under close follow-up.

**Conclusion:**

This case highlights the importance of vigilance for rare metastatic sites, such as the adrenal gland, in patients with advanced gastric cancer even after standardized postoperative surveillance. Systemic chemotherapy combined with immunotherapy may represent an effective treatment strategy for selected patients with metastatic gastric cancer.

## Introduction

Gastric cancer remains one of the most common gastrointestinal malignancies worldwide and is associated with a high rate of postoperative recurrence and metastasis (1). Common metastatic sites include the liver, peritoneum, lungs, and lymph nodes. Although previous studies have reported that approximately 16%–18% of patients with gastric cancer develop adrenal metastases (2), the majority of these cases occur in the setting of synchronous multiple metastases involving other organs (3). Solitary adrenal metastasis occurring after curative gastrectomy is particularly uncommon and poses diagnostic and therapeutic challenges.

Due to the absence of specific clinical symptoms and the limited sensitivity of routine laboratory tests and tumor markers, adrenal metastases are frequently missed during follow-up (4). Consequently, even in patients undergoing standardized postoperative surveillance and imaging follow-up, adrenal metastatic lesions may still emerge abruptly on imaging studies within a short period of time. Here, we report a rare case of solitary adrenal metastatic adenocarcinoma detected 21 months after radical gastrectomy for advanced gastric cancer and discuss the diagnostic approach and therapeutic strategy in light of current literature.

## Case description

### Patient information

A 69-year-old male was admitted to our institution with a two-week history of intermittent pain in the left upper abdomen. He had undergone radical gastrectomy for esophagogastric junction cancer 21 months earlier. Histopathological examination revealed diffuse infiltrative mucinous adenocarcinoma with components of signet-ring cell carcinoma and neuroendocrine carcinoma (pT4N3Mx). Surgical margins were negative, and metastatic lymph nodes were identified. The patient had completed six cycles of adjuvant chemotherapy with S-1 plus oxaliplatin. He had no significant family history of malignancy and no known genetic disorders. The patient should adhere to follow-up examinations every 3–6 months postoperatively. His postoperative follow-up had been unremarkable until the current presentation. Physical examination revealed mild tenderness in the left upper abdomen without palpable masses. No signs of endocrine dysfunction were observed. Vital signs were stable, and no other abnormal findings were noted.

### Diagnostic assessment

**FIGURE 1:**
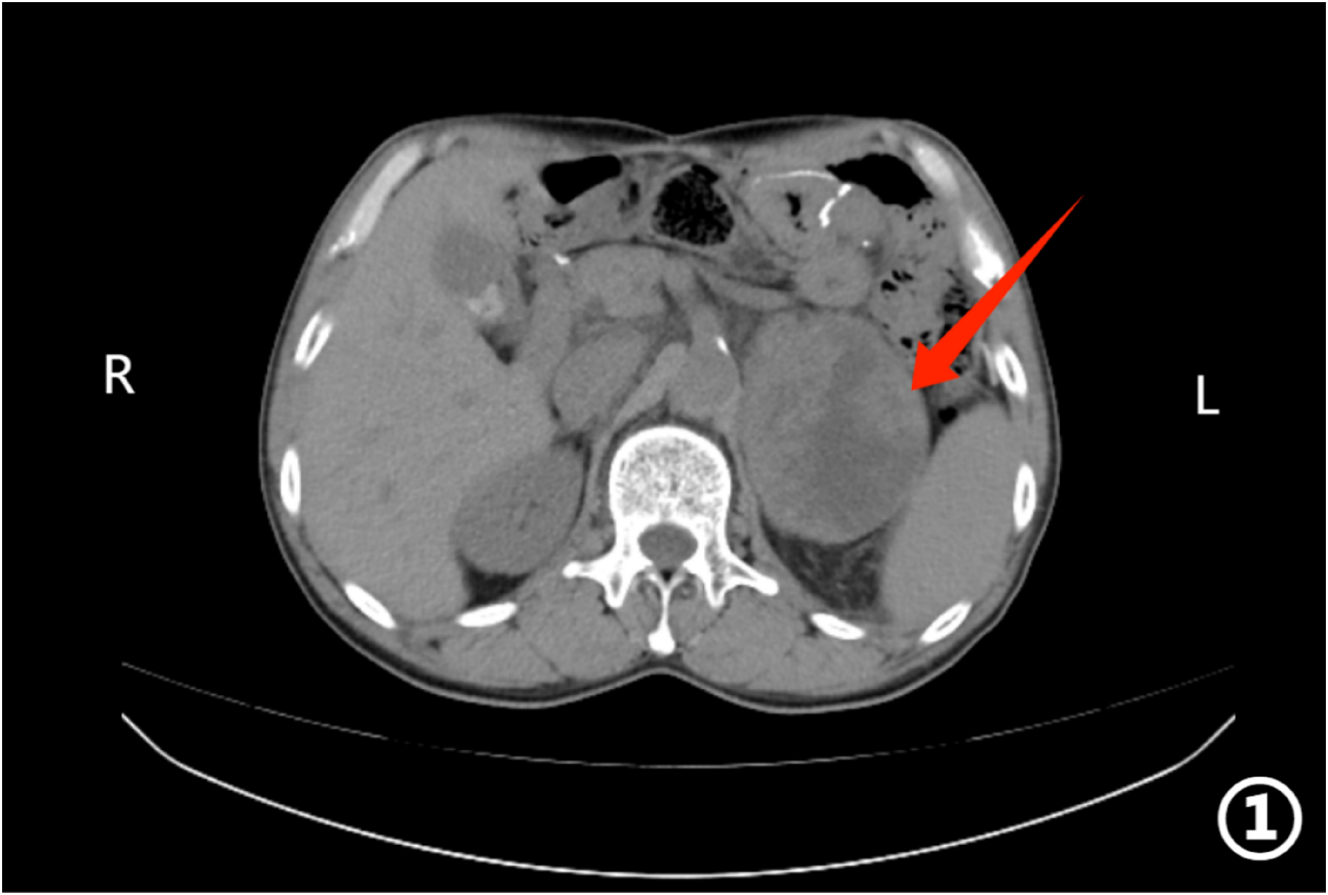
Axial abdominal CT image showing a large area of low-density shadow in the left upper abdomen within the splenic region (red arrow).

**FIGURE 2:**
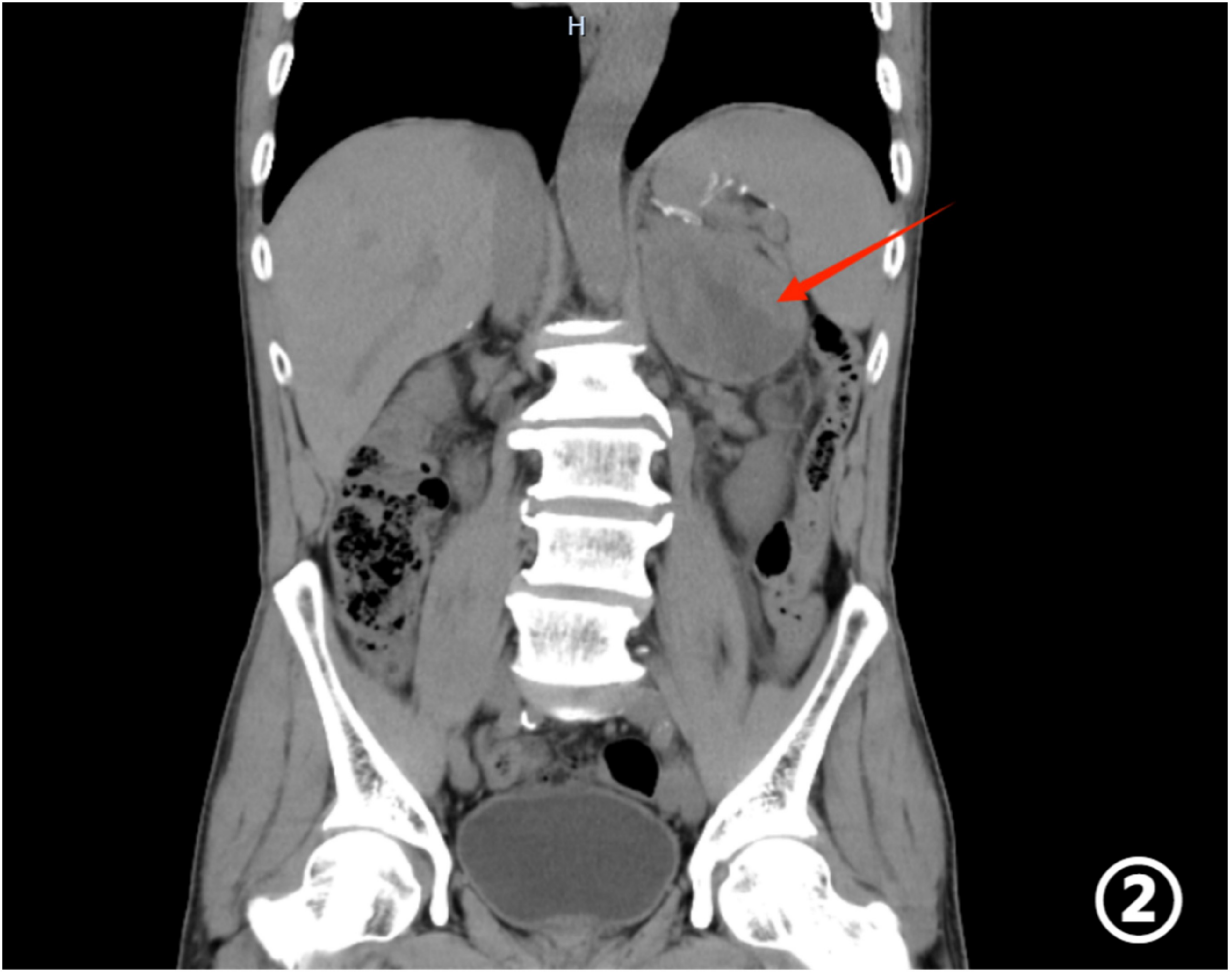
Abdominal CT coronal image: The red arrow points to the spleen area in the left upper abdomen. In the left adrenal region, there is a large soft tissue mass, with multiple nodular shadows visible around it.

Contrast-enhanced abdominal CT revealed a solitary mass in the left adrenal gland with irregular margins, showing a CT attenuation value of approximately 35 HU (Figures 1, 2). Laboratory tests demonstrated markedly elevated tumor markers, including CA72-4, CA19-9, and CEA. Before the procedure, we performed a comprehensive hormonal evaluation, including plasma free metanephrines, 24-h urinary catecholamines, serum cortisol, plasma aldosterone/renin ratio. All results were within normal ranges and showed no evidence of functional adrenal tumor. Differential diagnoses included adrenal adenoma, pheochromocytoma, primary adrenal carcinoma, and metastatic disease.

**FIGURE 3:**
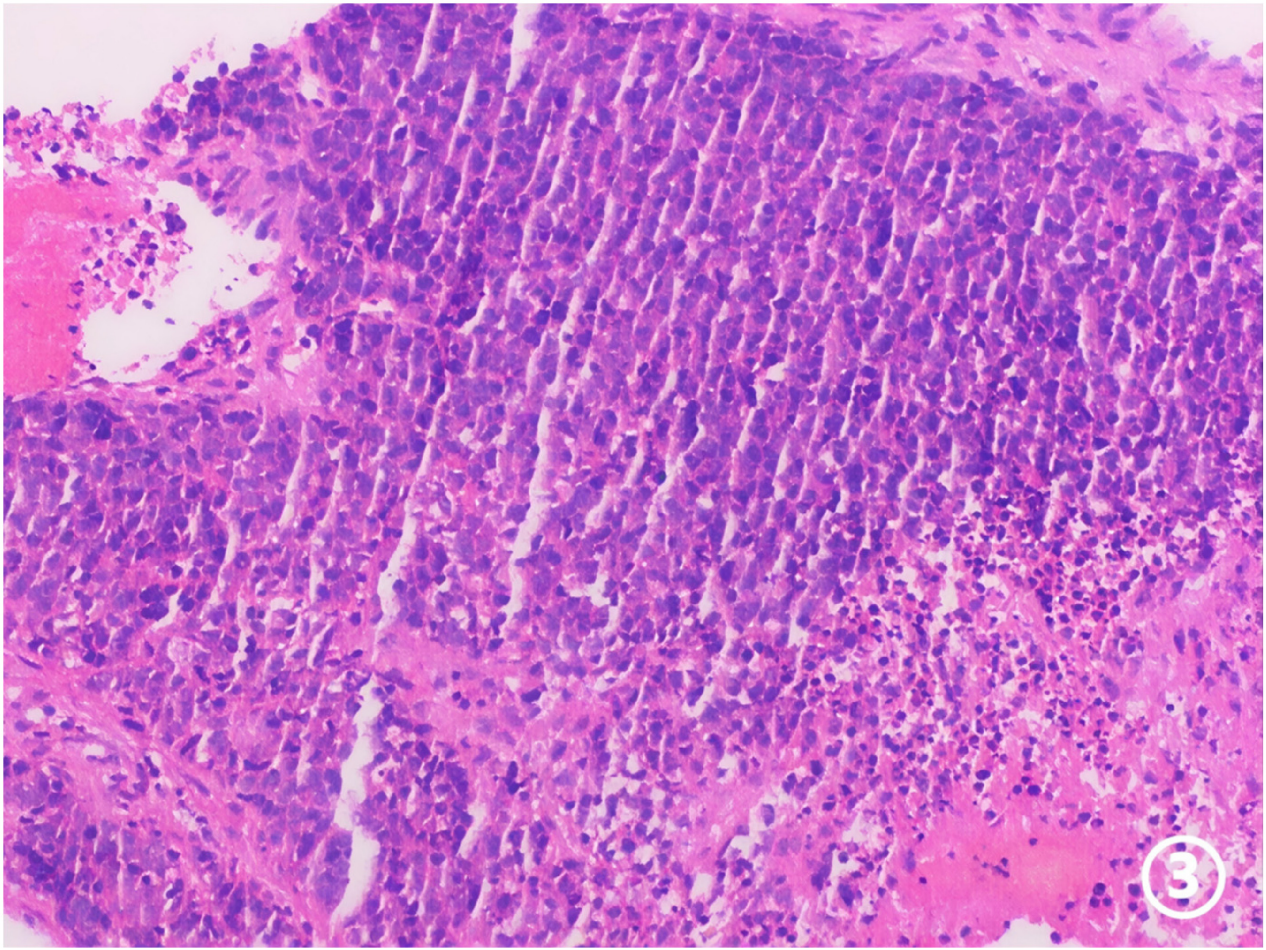
Pathological examination of the left kidney biopsy specimen: Within the fibrous stroma, numerous nuclei exhibit atypia, enlargement, and dense chromatin. The cell morphology is round or oval in shape. HE ×100.

**FIGURE 4:**
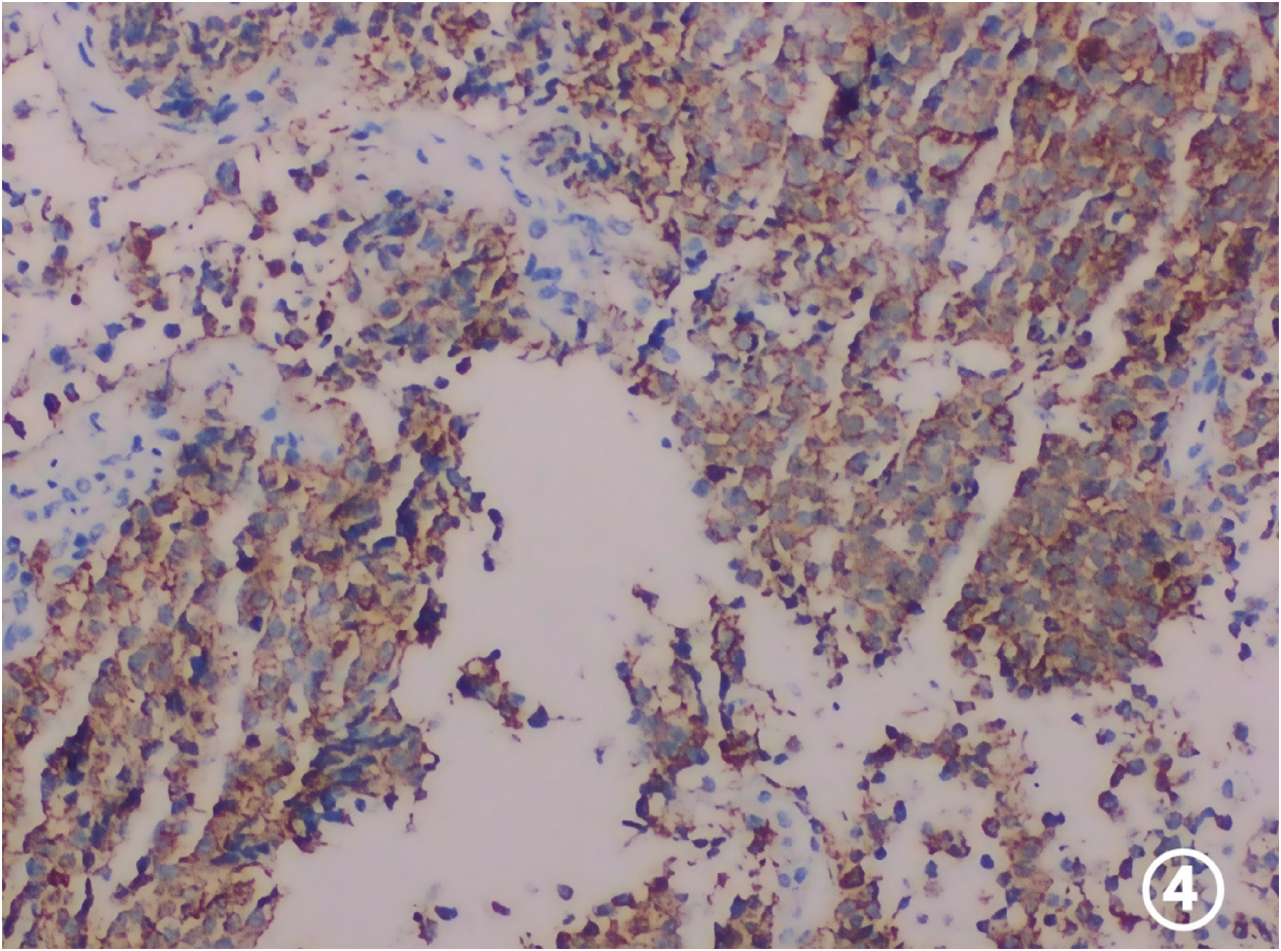
Immunohistochemistry showing CKPan positivity (+).

Ultrasound-guided core needle biopsy confirmed infiltrative metastatic adenocarcinoma (Figure 3). Immunohistochemical analysis supported a gastric origin (Figure 4), establishing the diagnosis of solitary adrenal metastasis from gastric cancer.

### Therapeutic intervention

Considering the advanced pathological features of the primary tumor and the high risk of systemic micrometastases, systemic therapy was selected. The patient received chemotherapy with the SOX regimen (S-1 plus oxaliplatin) combined with the PD-1 inhibitor tislelizumab. Supportive care, including antiemetic therapy and nutritional support, was provided.

### Follow-up and outcomes

After the first cycle of treatment, the patient’s general condition improved, with stable appetite and sleep. Intermittent left upper abdominal pain persisted but was tolerable. No severe adverse events were observed. The patient remains under close follow-up, with ongoing assessment of imaging findings and tumor markers.

## Discussion

Postoperative recurrence and metastasis remain the major determinants of prognosis in patients with gastric cancer (5), with the most common metastatic sites including the liver, peritoneum, and lymph nodes. The adrenal gland is a relatively uncommon target organ for gastric cancer metastasis, and clinical reports are limited. Autopsy studies have shown that the incidence of adrenal metastasis from gastric cancer ranges from approximately 16% to 18% (2); however, due to its insidious onset, the antemortem diagnostic rate in clinical practice is only 0.8%–4.6% (6). In most cases, adrenal metastasis occurs as part of widespread systemic dissemination in advanced gastric cancer, whereas solitary adrenal metastasis is particularly rare.

The mechanism underlying adrenal metastasis is believed to be closely related to the gland’s rich vascular supply (7). Tumor cells may disseminate hematogenously to the adrenal gland via the abdominal aorta or renal arteries. The sinusoidal structure of adrenal blood flow facilitates tumor cell arrest and implantation (8). In addition, retrograde lymphatic spread has also been proposed as a possible metastatic pathway. In the present case, a solitary left adrenal mass was detected 21 months after radical gastrectomy. Combined with the postoperative pathological stage (pT4N3Mx), this finding suggests that patients with locally advanced disease and a high lymph node metastatic burden remain at risk for late hematogenous metastasis, even after standardized adjuvant chemotherapy.

Early-stage adrenal metastasis from gastric cancer usually lacks specific clinical manifestations and is most often detected incidentally during routine follow-up computed tomography (CT) examinations (9). As the tumor enlarges, compression of adjacent organs or invasion of retroperitoneal nerves may result in lumbar or abdominal pain, as observed in this patient, who presented with left upper abdominal pain. In rare cases, bilateral adrenal involvement may lead to adrenal insufficiency and manifestations of Addison’s disease. CT is the preferred initial imaging modality, and metastatic adrenal lesions typically appear as irregularly marginated masses with heterogeneous density and marked enhancement. Differential diagnoses include primary adrenal adenoma, pheochromocytoma, and adrenocortical carcinoma (10).

Elevated tumor markers, such as carcinoembryonic antigen (CEA), carbohydrate antigen 19-9 (CA19-9), and carbohydrate antigen 72-4 (CA72-4), may provide important diagnostic clues. In this case, multiple tumor markers were markedly elevated and were consistent with the imaging findings. Nevertheless, histopathological examination remains the gold standard for diagnosis. Given the complex pathological features of the primary tumor in this patient—including signet-ring cell carcinoma and neuroendocrine carcinoma components with high malignant potential—image-guided biopsy not only confirmed the diagnosis of metastatic adenocarcinoma but also, through immunohistochemical analysis, established its gastric origin. This effectively excluded the possibility of a second primary malignancy and provided a solid basis for subsequent therapeutic decision-making.

According to the 2022 gastric cancer treatment guidelines, therapeutic strategies for patients with distant metastasis should be individualized based on performance status, number of metastatic lesions, and control of the primary tumor. For solitary adrenal metastasis, some authors advocate adrenalectomy, suggesting that R0 resection may prolong survival in selected patients (11). However, in the present case, the advanced pathological stage of the primary tumor (T4N3), the presence of unfavorable histological components such as neuroendocrine carcinoma and signet-ring cell carcinoma, and a high Ki-67 index (approximately 70%) indicated highly aggressive tumor biology and a substantial risk of occult systemic micrometastases. Under these circumstances, local surgical intervention alone was unlikely to provide meaningful benefit, and systemic therapy was considered a more appropriate initial treatment strategy.

The patient was therefore treated with the SOX regimen (tegafur–gimeracil–oteracil plus oxaliplatin) combined with the PD-1 inhibitor tislelizumab (Toripalimab). This approach aims to alleviate tumor-related symptoms, improve quality of life, and potentially prolong survival in patients with unresectable, recurrent, or metastatic gastric cancer who have good performance status and preserved organ function. In recent years, immune checkpoint inhibitors targeting the PD-1/PD-L1 pathway have achieved significant breakthroughs in the treatment of advanced gastric cancer (12, 13). Large clinical trials, including CheckMate-649, KEYNOTE-062, and ORIENT-16, have demonstrated that immunotherapy combined with chemotherapy provides substantial survival benefits in both HER2-negative and HER2-positive gastric cancer, significantly improving overall survival (OS) and progression-free survival (PFS). In this case, chemotherapy was initiated promptly to target highly proliferative tumor cells, while PD-1 blockade was used to activate antitumor immune responses, with the goal of achieving synergistic tumor control.

The primary tumor in this patient contained both neuroendocrine and signet-ring cell components, a mixed histological subtype known for its aggressive behavior and poor prognosis. Such tumors are prone to vascular tumor emboli formation and neural invasion (14), which likely explains the occurrence of hematogenous metastasis despite completion of full-course adjuvant chemotherapy. Although the patient’s general condition remained stable after one treatment cycle, adrenal metastasis often indicates systemic micrometastatic dissemination and is generally associated with an unfavorable prognosis. Close monitoring of imaging findings and tumor marker levels is therefore essential to evaluate treatment response. If significant tumor regression is observed after three to four cycles of systemic therapy, with no evidence of new metastatic lesions and sustained good performance status, a multidisciplinary team (MDT) may reconsider the feasibility of salvage adrenalectomy to potentially achieve a disease-free status.

## Conclusion

Adrenal metastasis from gastric cancer is rare and clinically insidious. Long-term postoperative surveillance is particularly important for patients with advanced pathological stage and highly aggressive histological features. CT imaging combined with tumor marker monitoring remains the cornerstone of follow-up, while image-guided biopsy is critical for definitive diagnosis. Systemic chemotherapy combined with immunotherapy showed potential efficacy and tolerability in this specific case and warrants further investigation in larger cohorts, might offer a promising therapeutic option for patients with recurrent or metastatic gastric cancer; however, the optimal treatment strategy should be determined on an individualized basis.

## Data Availability

The original contributions presented in this study are included in this article/supplementary material, further inquiries can be directed to the corresponding author.
